# Availability of essential medicines, progress and regional distribution in China: a systematic review and meta-analysis

**DOI:** 10.3389/fpubh.2023.1149838

**Published:** 2023-04-25

**Authors:** Miao Zhang, Kun Zou, Zheng Liu, Dan Liu, Xiuli Wang, Yuqing Shi, Zhe Chen, Xiao Cheng, Bingchen Lang, Hailong Li, Linan Zeng, Yong Tang, Shaoyang Zhao, Yongmu Jiang, Imti Choonara, Lingli Zhang

**Affiliations:** ^1^Department of Pharmacy, West China Second University Hospital, Sichuan University, Chengdu, China; ^2^Evidence-Based Pharmacy Center, West China Second University Hospital, Sichuan University, Chengdu, China; ^3^National Medical Products Administration (NMPA) Key Laboratory for Technical Research on Drug Products *In Vitro* and *In Vivo* Correlation, Chengdu, China; ^4^Key Laboratory of Birth Defects and Related Diseases of Women and Children, Sichuan University, Ministry of Education, Chengdu, China; ^5^West China School of Pharmacy, Sichuan University, Chengdu, China; ^6^West China School of Medicine, Sichuan University, Chengdu, China; ^7^Healthcare Evaluation and Organizational Analysis (HEOA) Group, West China School of Public Health and West China Fourth Hospital, Sichuan University, Chengdu, China; ^8^School of Economics, Sichuan University, Chengdu, China; ^9^Academic Division of Child Health, University of Nottingham, Derbyshire Children's Hospital, Derby, United Kingdom; ^10^Chinese Evidence-Based Medicine Center, West China Hospital, Sichuan University, Chengdu, China

**Keywords:** essential medicines, availability, China, regional distribution, systematic review

## Abstract

**Background:**

Essential medicines are the backbone of healthcare and meet the priority healthcare needs of the population. However, approximately one-third of the global population does not have access to essential medicines. Although China formulated essential medicine policies in 2009, the progress of availability of essential medicines and regional variations remains unknown. Therefore, this study was conducted to evaluate the availability of essential medicines, their progress, and regional distribution in China in the last decade.

**Methods:**

We searched eight databases from their inception to February 2022, relevant websites, and reference lists of included studies. Two reviewers selected studies, extracted data, and evaluated the risk of bias independently. Meta-analyses were performed to quantify the availability of essential medicines, their progress, and regional distribution.

**Results:**

Overall 36 cross-sectional studies conducted from 2009 to 2019 were included, with regional data for 14 provinces. The availability of essential medicines in 2015–2019 [28.1%, 95% confidence interval (CI): 26.4–29.9%] was similar to that in 2009–2014 (29.4%, 95% CI: 27.5–31.3%); lower in the Western region (19.8%, 95% CI: 18.1–21.5%) than Eastern (33.8%, 95% CI: 31.6–36.1%) and Central region (34.5%, 95% CI: 30.6–38.5%); very low for 8 Anatomical Therapeutic Chemical (ATC) categories (57.1%), and low for 5 categories (35.7%) among all ATC groups.

**Conclusion:**

The availability of essential medicines in China is low compared with the World Health Organization goal, has not changed much in the last decade, is unequal across regions, and lacks data for half of provinces. For policy-making, the monitoring system of the availability of essential medicines is to be strengthened to enable long-term surveillance, especially in provinces where the data has been missing. Meanwhile, Joint efforts from all stakeholders are warranted to improve the availability of essential medicines in China toward the universal health coverage target.

**Systematic review registration:**

https://www.crd.york.ac.uk/PROSPERO/display_record.php?RecordID=315267, identifier: PROSPERO CRD42022315267.

## 1. Introduction

Access to essential medicines is a vital component of the millennium development goals (MDGs), sustainable development goals (SDGs) and universal health coverage (UHC) (1–3). The World Health Organization (WHO) defined essential medicines as those can meet basic medical and healthcare needs, have appropriate dosage forms, guaranteed supply, can be equipped at the grass-roots level, and can be equitably accessed by the people (4). Since 1977, WHO has been updating essential medicines list every 2 years, with the latest 22nd version in 2021 (5). Currently, the list forms an integral part of national essential medicines policies in 146 countries guiding the selection of drugs based on public health relevance, efficacy, safety, and cost (6). However, studies have shown that millions of people around the world face illness, disability, and death every year because of poor access to medicines (7, 8). Approximately one-third of the global population does not have access to essential medicines. In 2012, a survey performed by WHO estimated that more than 10 million deaths around the world could be avoided every year by providing essential medicines through the effective National Essential Medicines Policy (NEMP) (9). To facilitate and promote monitoring the progress of the availability of essential medicines and the national essential medicine system (NEMS), the WHO and Health Action International (HAI) jointly developed the WHO/HAI standardized method, which provided a unified method for countries and organizations to investigate the availability, price, and affordability of essential medicines (10).

In 2009, China initialized the national essential medicine system, as one of the five key components of the “new health reform” to improve the medicine supply system and ensure the safe use of medicines in the population (11). Since the new health reform in China in 2009, several studies have been conducted to examine the availability of essential medicines in China (12–19). However, several key questions have not been fully addressed. Firstly, nationwide studies of the availability of essential medicines were lacking, as most of these studies were conducted in single or several provinces only (20). Secondly, studies of the findings on its secular trend have been inconsistent (17–19, 21–23). For example, Zhu et al. (18) and Wei et al. (19) showed that the availability of essential medicines in 2016 was lower than that in 2012. In contrast, a study found that the availability of essential medicines increased from 2010 to 2014 (17). A thorough evaluation on the secular progress and geographical distribution of the availability of essential medicines in China could enable benchmarking and provide vital evidence for policy-making on the NEMS for years to come. However, to our knowledge, no such study has been conducted in China since its 2009 health reform.

Therefore, this study was conducted to systematically evaluate the availability of essential medicines, their secular progress, and regional distribution in China in the last decade, to provide evidence and support policy-making of the NEMS to improve the availability of essential medicines toward universal health coverage.

## 2. Methods

This systematic review and meta-analysis was reported in accordance with the preferred reporting items for systematic review and meta-analysis (PRISMA) (24), and was registered in PROSPERO (CRD42022315267).

### 2.1. Search strategy

We searched literature databases of PubMed, Embase (Ovid), the Cochrane Library, Web of Science, China National Knowledge Infrastructure (CNKI), WanFang database, VIP database, and Chinese Biomedical Literature database (CBM) from their inceptions to February 2022. We also searched the websites the of WHO and International Pharmaceutical Federation (FIP) and manually checked reference lists of included studies and relevant published reviews. The search terms included: essential drug, essential medicine, China, Chinese, etc. The search strategy in PubMed is presented in Supplementary Table S1.

### 2.2. Eligibility criteria

Primary studies were included if they had participants of medical institutions or pharmacies, public or private, that may provide essential medicines in mainland China; outcome of availability of essential medicines (frequency and percentage); study design of the cross-sectional study, interrupted times series study, uncontrolled before-after study, and controlled before-after study. Publications in English or Chinese were included. Studies were excluded if they were duplicate publications or their full texts were not available.

### 2.3. Study selection and data extraction

Two reviewers (MZ and ZL) selected studies and extracted data independently. The following data were extracted: (1) general information of the study: title, first author, year of publication, and study design; (2) characteristic of studies: study area, survey time, survey method, number of essential medicines investigated, number of medical organizations, and names of investigated medicines; (3) outcome: availability rate of essential medicines. Reviewers resolved disagreements by discussion to consensus, and if necessary, by consulting a third reviewer (KZ).

### 2.4. Risk of bias assessment

Two reviewers (MZ and ZL) evaluated the risk of bias of cross-sectional studies independently using the Joanna Briggs Institute (JBI) Critical Appraisal Tools (25). The tool consists of nine items in terms of sampling methods, research objects, data collection, and analysis methods. Each item was determined by yes, no, unclear, and not applicable. For the overall quality rating of a study, more than 6 scores were considered as high, 4–6 scores as moderate, and < 4 scores as low quality (25). The risk of bias of interrupted times series study, uncontrolled before-after study, and controlled before-after study was assessed using the Cochrane Effective Practice and Organization of Care (EPOC) criteria (26). Disagreements were resolved by discussion to consensus, and if necessary, by consulting a third reviewer (KZ).

### 2.5. Outcome measurement

The availability of essential medicines was calculated as the percentage (%) of the number of institutions equipped with essential medicines to the number of institutions surveyed. The availability of essential medicines was classified as very low if the percentage was < 30%, low if it was 30–49%, fairly high if it was 50–80%, and high if it was more than 80% (27).

### 2.6. Statistical analysis

The pooled availability of essential medicines was estimated using percentage and its 95% CI. Heterogeneity was evaluated using *I*^2^ and Chi-square (χ^2^) test. If heterogeneity was significant (*I*^2^ > 50%), the random effect model was used, otherwise, the fixed effect model was used. The overall availability rates of essential medicines in China, and that from 2009 to 2014 and from 2015 to 2019 were estimated. Subgroup analyses were conducted by regions in mainland China (Eastern region including 11 provinces or municipalities, Central region including 8 provinces or autonomous regions, and Western region including 12 provinces, municipality or autonomous regions) (28), and provinces to examine its geographic distribution across China, and by Anatomical Therapeutic Chemical (ATC) Classification of medicines.

## 3. Results

### 3.1. Literature search and study selection

**Figure 1:**
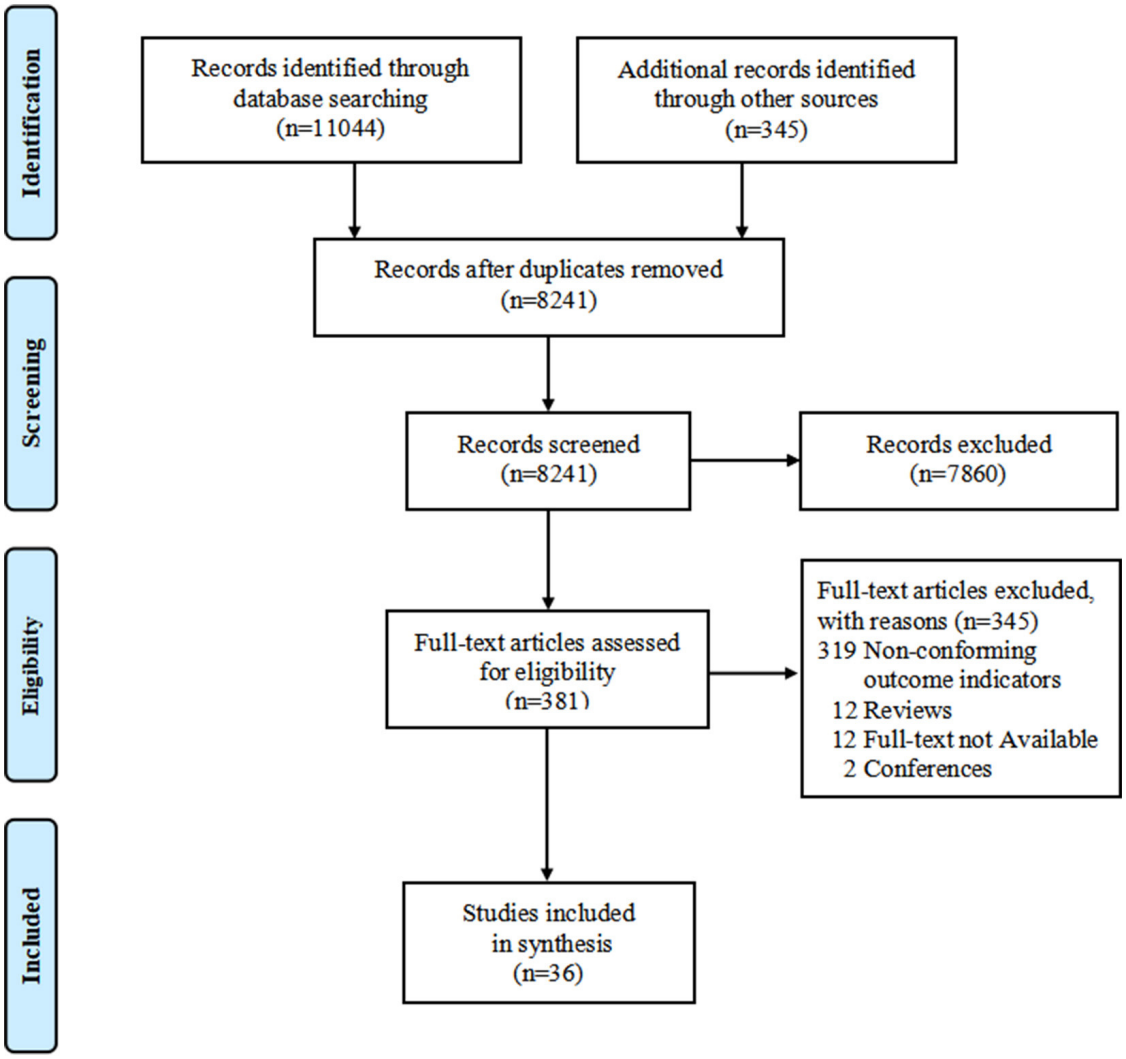
PRISMA flow chart of study selection.

A total of 11,389 records were identified by the initial search. After removing duplicates and irrelevant records by screening for titles and abstracts, 381 studies were assessed for eligibility at full-text screening. Eventually, 36 studies were included in this systematic review (13, 16–23, 29–55). The study selection process is shown in Figure 1.

### 3.2. Characteristics of included studies

The characteristics of the included studies are presented in Table 1. The included were all cross-sectional studies, which were conducted from 2009 to 2019. Among them, 15 studies (41.7%) were conducted in the Eastern region, 8 studies (22%) in the Western region, 4 studies (11%) in the Central region, and the remaining 9 studies (25%) were conducted across several provinces or nationwide. Among them, 33 studies (92%) adopted the WHO/HAI standardized methodology, while the investigation method was unclear in 3 studies. However, they all adopted the definition of availability of essential medicines according to the WHO/HAI methods, and therefore, were also included in the analyses. All studies selected investigated medicines based on the WHO Model List of Essential Medicines (22nd List) and China National Essential Medicines List (2018) (56, 57). The median number of investigated essential medicines was 28 (ranging from 3 to 121). The list of essential medicines investigated in included studies is presented in Supplementary Table S2.

**Table 1:** Characteristics of included studies.

**Study ID**.	**Study time**	**Study area**	**Region**	**Methods**	**Number of medical organizations**	**Number of essential medicines**
Zhu et al. (29)	2013–2019	Jiangsu	Eastern region	WHO/HAI	41	12
Wang et al. (23)	2016, 2018	Jiangsu	Eastern region	WHO/HAI	56	3
Zhang et al. (20)	2019	Jiangsu	Eastern region	WHO/HAI	13	35
Wang et al. (31)	2019	Shandong	Eastern region	WHO/HAI	150	30
Dai et al. (32)	2017	China (Zhejiang, Fujian, Jiangxi, Jiangsu, Anhui, Guangdong, Henan, Yunnan, Guizhou, Sichuan, Shaanxi, Hebei, Shandong, Liaoning, Guangxi, Xinjiang, Shanghai)	Nationwide	WHO/HAI	55	42
Zhang et al. (30)	2018	China (The province was not specified)	Nationwide	Unclear	2,243	28
Dong et al. (35)	2018	Zhejiang	Eastern region	WHO/HAI	60	12
Yang et al. (33)	2018	China (Shandong, Hubei, Henan, Shaanxi, Yunnan)	Nationwide	WHO/HAI	519	12
Xu et al. (34)	2015	Anhui	Central region	WHO/HAI	143	13
Jiang et al. (36)	2017	Liaoning	Eastern region	WHO/HAI	76	25
Li et al. (16)	2016	Shaanxi	Western region	WHO/HAI	21	8
Wei et al. (19)	2012, 2016	NR (The province was not specified)	Eastern region	WHO/HAI	1725	49
Zhu et al. (18)	2012, 2016	Jiangsu	Eastern region	WHO/HAI	70	40
Yang et al. (37)	2015	Shaanxi	Western region	WHO/HAI	144	8
Sun et al. (41)	2017	Jiangsu	Eastern region	WHO/HAI	60	40
Xi et al. (39)	2017	China (Shanghai, Jiangsu, Shandong, Ningxia, Jiangxi, Henan)	Nationwide	WHO/HAI	24	50
Li et al. (38)	2016	China (Henan, Fujian, Xinjiang, Gansu, Nei Monggol, Hunan, Shaanxi, Shandong, Anhui, Zhejiang, Shanxi, Jilin, Liaoning, Guangdong, Ningxia, Jiangsu, Chongqing, Yunnan, Beijing)	Nationwide	WHO/HAI	55	121
Gong et al. (42)	2016	Hubei	Central region	WHO/HAI	34	20
Wu et al. (40)	2016	Hubei	Central region	WHO/HAI	33	16
Guan et al. (21)	2011–2016	China (28 provinces, municipalities and autonomous regions except Qinghai, Xizang, Hainan)	Nationwide	WHO/HAI	1,159	13
Song et al. (22)	2009–2011	China (Shandong, Zhejiang, Anhui, Ningxia)	Nationwide	WHO/HAI	146	NR
Su et al. (43)	2016–2017	China (31 mainland provinces, municipalities and autonomous regions)	Nationwide	Unclear	3,362	62
Liu et al. (13)	2016	Hubei	Central region	WHO/HAI	60	5
Xie et al. (44)	2015	Shanghai	Eastern region	WHO/HAI	13	30
Shang et al. (45)	2013	Beijing	Eastern region	Unclear	1,585	9
Wu et al. (17)	2010, 2012, 2014	Shaanxi	Western region	WHO/HAI	374	44
Zhang and Li (46)	2013	Jiangsu	Eastern region	WHO/HAI	60	24
Xi et al. (47)	2013	Jiangsu	Eastern region	WHO/HAI	63	30
Jiang et al. (48)	2012	Shaanxi	Western region	WHO/HAI	240	35
Wang et al. (49)	2012	Shaanxi	Western region	WHO/HAI	120	21
Wang et al. (50)	2011	Shaanxi	Western region	WHO/HAI	30	21
Jiang et al. (52)	2012	Shaanxi	Western region	WHO/HAI	240	38
Guan et al. (53)	2010	China (The province was not specified)	Nationwide	WHO/HAI	334	30
Yan et al. (51)	2010	Shaanxi	Western region	WHO/HAI	86	33
Li et al. (54)	2010	Zhejiang	Eastern region	WHO/HAI	17	14
Li (55)	2010	Guangdong	Eastern region	WHO/HAI	28	40

WHO/HAI, The World Health Organization/Health Action International; NR, Not reported.

### 3.3. Risk of bias assessment

Among the 36 studies, 26 studies were assessed as high quality, and the remaining 10 studies were of moderate quality (Supplementary Table S3).

### 3.4. Availability of essential medicines in China from 2009 to 2019

A total of 36 studies reported the availability of essential medicines in China. Overall, the availability of essential medicines was 28.8% (95% CI: 27.5–30.1%) from 2009 to 2019. Detailed availability of the essential medicines by ATC is presented in Supplementary Table S4.

#### 3.4.1. Secular trend of availability of essential medicines in China between 2009–2014 and 2015–2019

**Figure 2:**
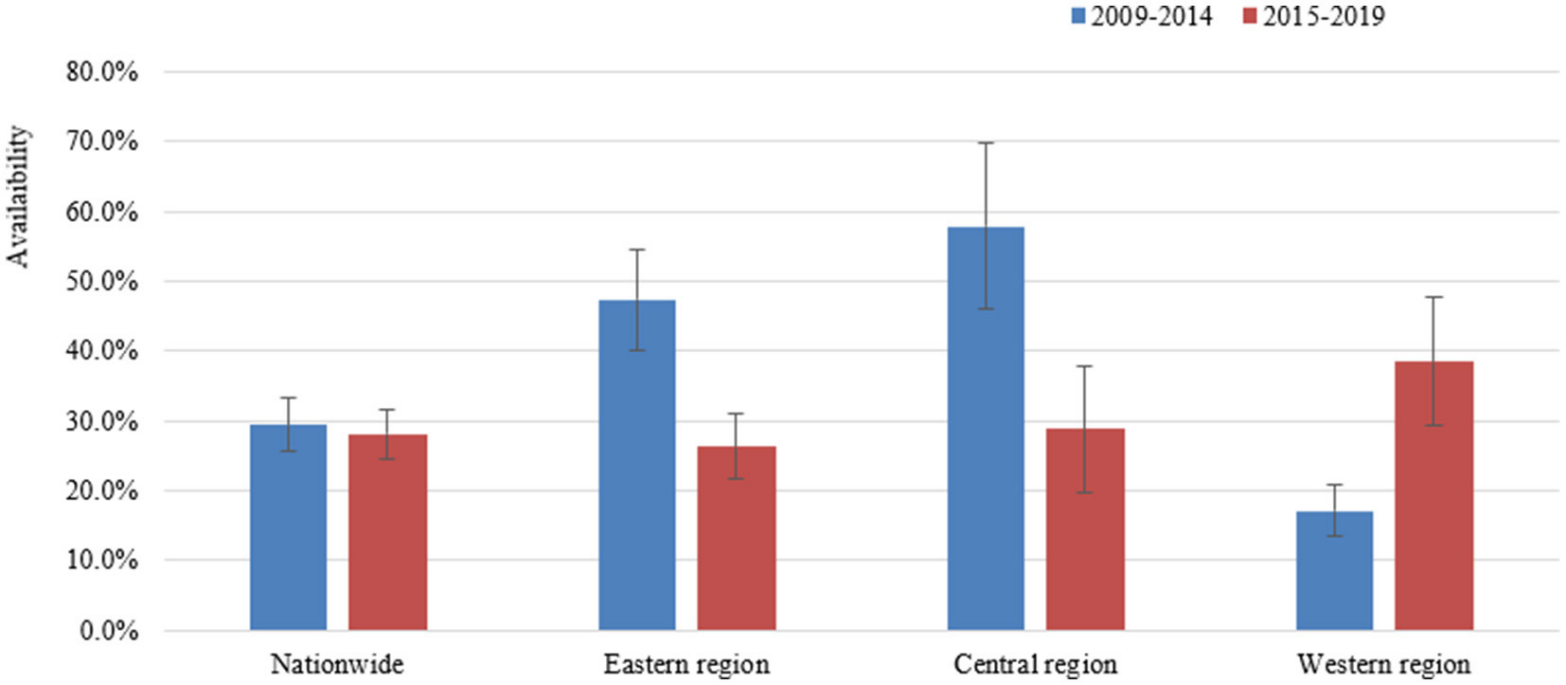
The availability of essential medicines in China.

The changes of the availability of essential medicines in China during the two periods are shown in Figure 2. A total of 17 studies reported the availability from 2009 to 2014, and 23 studies from 2015 to 2019. The overall availability of essential medicines in China during the two periods was similar, which was 29.4% (95% CI: 27.5–31.3%) from 2009 to 2014 and 28.1% (95% CI: 26.4–29.9%) from 2015 to 2019. Detailed availability of the essential medicines by ATC between the two periods is presented in Supplementary Tables S5, S6.

#### 3.4.2. Availability of essential medicines by regions

The number of studies reported the availability of essential medicines for the Eastern, Central and Western regions was 20, 9, and 13, respectively. From 2009 to 2019, the overall availability of essential medicines in the Western regions (19.8%, 95% CI: 18.1–21.5%) was lower than that in the Eastern (33.8%, 95% CI: 31.6–36.1%) and Central regions (34.5%, 95% CI: 30.6–38.5%) (Supplementary Table S3). As regards to secular trend, the availability of essential medicines in the Eastern region and the Central region reduced notably from 2015 to 2019 to that from 2009 to 2014, while increasing substantially in the Western region from 17% during 2009–2014 to 38% from 2015 to 2020 (Figure 2; Supplementary Tables S5, S6).

#### 3.4.3. Availability of essential medicines by provinces

**Figure 3:**
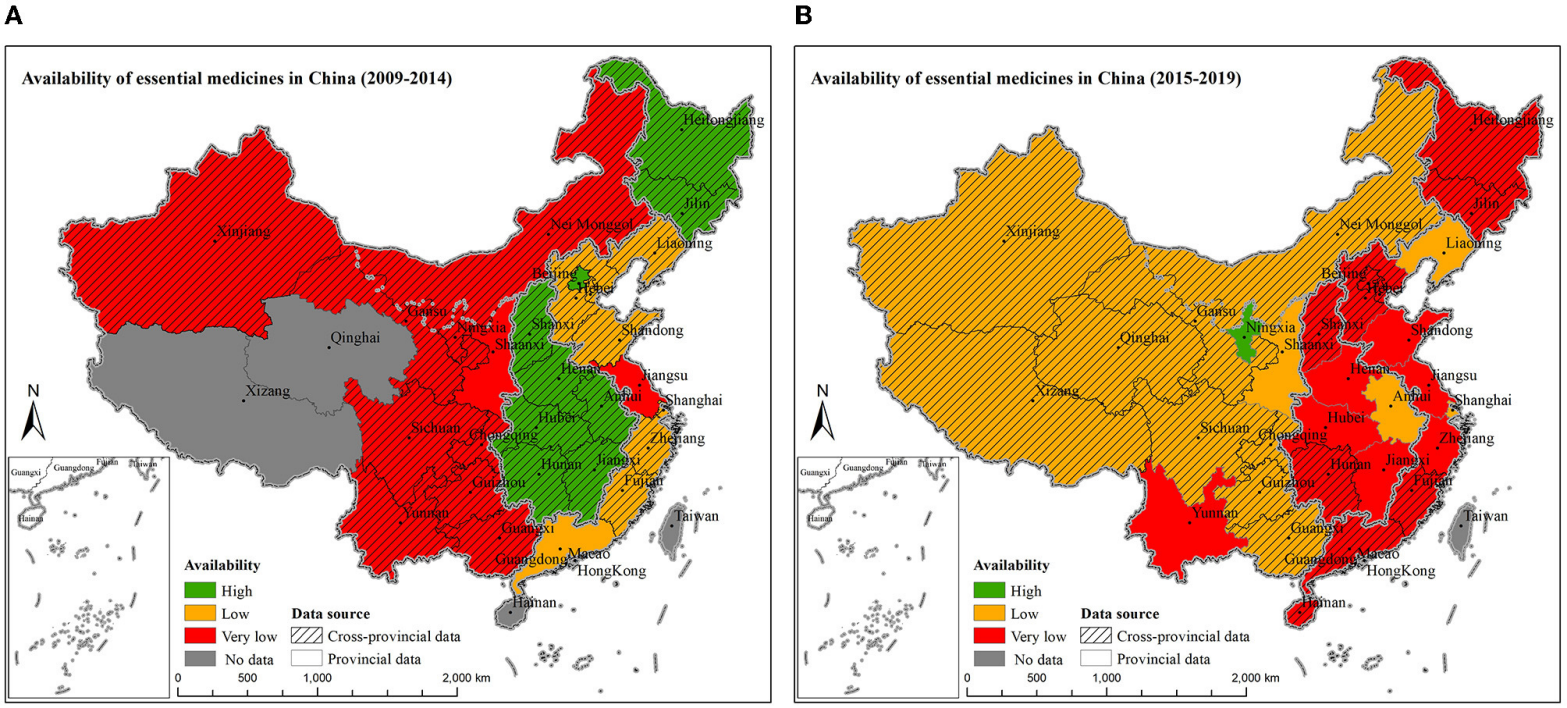
The availability of essential medicines by provinces from 2009 to 2014 **(A)** and from 2015 to 2019 **(B)**. Availability of essential medicine considered as: high > 80%, fairly high 50–80%, low 30–49%, very low < 30%. Methods for data manipulation for provinces with missing data in the map: if the province has been surveyed but the provincial data were not available, regional data (pooled estimate using meta-analysis) were used to fill in the data; if no survey has been conducted in the province at all, then it was labeled as no data. (From 2009 to 2014, provincial data were available for Beijing, Jiangsu, Guangdong, and Shaanxi; whereas, from 2015 to 2019, provincial data were available for Jiangsu, Liaoning, Shanghai, Zhejiang, Shandong, Anhui, Jiangxi, Henan, Hubei, Shaanxi, Yunnan, and Ningxia).

The variations of availability of essential medicines across provinces during the two time periods are shown in Figures 3A, B. Provincial data of the availability of essential medicines were only reported for 14 provinces, while the other 17 provinces neither conducted a survey nor reported the provincial data. Among them, 5 provinces (16.1%) had provincial data of availability of essential medicines from 2009 to 2014, and 12 provinces (38.7%) had provincial data from 2015 to 2019.

From 2009 to 2019, the three provinces with the lowest overall availability of essential medicines were Yunnan (19.4%, 95% CI: 6.3–3.2%), Shaanxi (19.8%, 95% CI: 18.0–21.6%), and Jiangsu (22.3%, 95% CI: 19.4–25.1%). More detailed availability of essential medicines in different provinces from 2009 to 2014 and from 2015 to 2019 were presented in Supplementary Tables S5, S6.

#### 3.4.4. Availability of essential medicines by ATC categories

**Figure 4:**
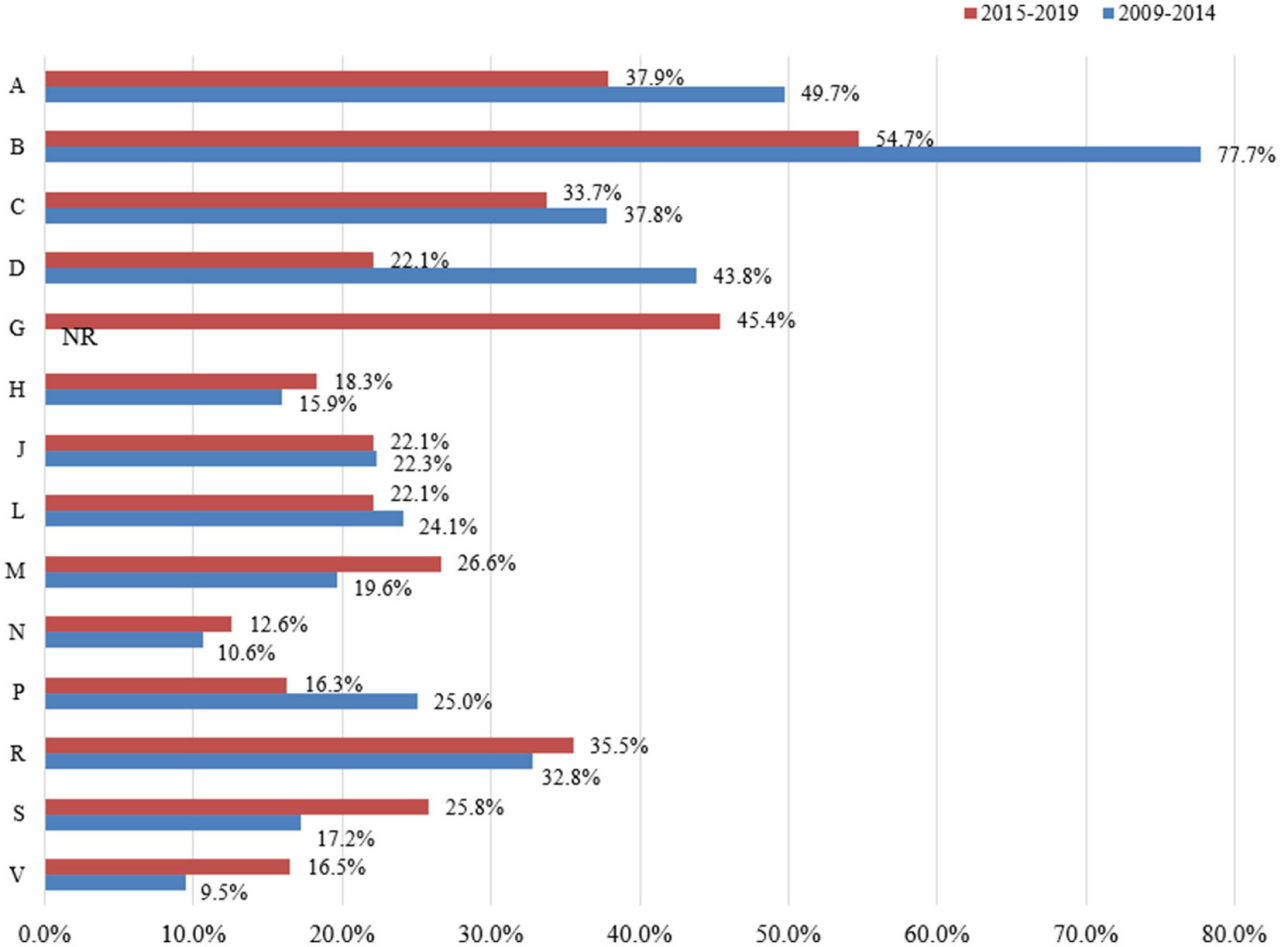
Changes in the availability of essential medicines between different categories based on ATC (2009–2014 vs. 2015–2019). G: Not reported the availability of Genito urinary system and sex hormones from 2009 to 2014. A: Alimentary tract and metabolism; B: Blood and blood forming organs; C: Cardiovascular system; D: Dermatologicals; G: Genito urinary system and sex hormones; H: Systemic hormonal preparations, excl. sex hormones and insulins; J: Antiinfectives for systemic use; L: Antineoplastic and immunomodulating agents; M: Musculo-skeletal system; N: Nervous system; P: Antiparasitic products, insecticides and repellents; R: Respiratory system; S: Sensory organs: V: Various.

Thirteen categories of essential medicines were investigated from 2009 to 2014, and 14 categories were investigated from 2015 to 2019. Changes in the availability of essential medicines by ATC categories in the two periods are presented in Figure 4.

From 2009 to 2014, the number of ATC categories of essential medicines with fairly high, low, and very low availability were 1, 4, and 8, respectively, while no data was available for the genito urinary system and sex hormones. From 2015 to 2019, the number of ATC categories of essential medicines with fairly high, low, and very low availability were 1, 4, and 9, respectively. From 2009 to 2014, the three ATC categories of essential medicines with the lowest availability were nervous system (10.6%, 95% CI: 8.1–13.4%), systemic hormonal preparations excluding sex hormones and insulins (15.9%, 95% CI: 2.8–34.6%), and sensory organs (17.2%, 95% CI: 7.0–30.5%). From 2015 to 2019, the three ATC categories of essential medicines with the lowest availability were nervous system (12.6%, 95% CI: 8.4–17.5%), antiparasitic products, insecticides and repellents (16.3%, 95% CI: 6.7–28.2%), and systemic hormonal preparations excluding sex hormones and insulins (18.3%, 95% CI: 6.6–33.5%). The three medicines with the lowest availability were similar to the national level, especially nervous system, which was very low (< 30%) in all regions (Supplementary Tables S3–S5).

## 4. Discussion

To our knowledge, this is the first systematic review and meta-analysis that has comprehensively evaluated the secular trend, regional and provincial distribution of the availability of essential medicines in China in the last decade. There are four important findings in this study. First, the availability of essential medicines was low in China from 2009 to 2019 with little overall change between 2009–2014 and 2015–2019, and much is to be done to achieve the goal of 80% availability suggested by the WHO. Second, the availability of essential medicines in the Western region was lower than that in the Eastern and Central regions. As regards to secular trend, the availability of essential medicines in the Eastern region and the Central region reduced slightly from 2015 to 2019 compared with that from 2009 to 2014, while increasing moderately in the Western region. Third, among 14 ATC categories of essential medicines, the availability was very low for 8 categories (57.1%), and low for 5 categories (35.7%) in most recent studies. Finally, the provincial data are only available for less than half provinces (14 provinces) while lacking for the others, as they were not surveyed at all or not reported, indicating substantial research gaps and needs.

Overall, our results revealed that the availability of essential medicines was still low in China according to the WHO availability goal, which was consistent with the findings of published studies. A national study found that the mean availability of essential medicines in China was low (4.29–43.75%) (33). Another national survey found that the overall availability of essential medicines for children in China was low (< 35%) (38). Internationally, Mahmić-Kaknjo et al. found that the availability of essential medicines is still suboptimal from 2003 to 2011 in low- and middle-income countries (58). It suggested that the availability of essential medicines in China is at the middle level among all low- and middle-income countries. There may be several reasons for the low availability of essential medicines in China. First, on the supply side, the low price and meager profit of essential medicines may lower the motivation of the production and distribution enterprises to produce and supply essential medicines (12, 59). Second, on the demand side, patients may be influenced by misunderstood beliefs that cheap medicines may not be as effective as those with high prices (60), which may also lead to a decrease in demand, production and supply of these cheap price essential medicines. Last, the WHO/HAI method has strict restrictions on dosage forms and specifications which may be different from that in the China market, and therefore, may underestimate the actual availability of those medicines (17).

We found the availability of essential medicine in China has changed little in the last decade. This finding was supported by several other studies. Guan et al. found that the nationwide availability was steady from 2011 to 2016 (21), and Song et al. showed that the availability of essential medicines did not change radically (22). The possible reason may be that the availability of essential medicines decreased in the Central and Eastern regions, while increasing in the Western region from 2009–2014 to 2015–2019. In addition, in this study, based on ATC categories, we found that the availability of some essential medicines has increased and that of others has decreased during the two periods. Since our study included all available studies of essential medicines across China from 2009 to 2019, it is more likely to reflect the overall trend of China. However, research on causes of the changes is lacking, as most studies on the availability of essential medicines only reflected its status quo but not examined its trend and reasons. Therefore, more studies are needed to understand why there has been little change in essential medicines over the past decade.

In our study, it is shown that the availability of essential medicines in the Western region was lower than in the Eastern region, which was consistent with findings from previous studies. Guan et al. and Su et al. both found that compared with the Eastern region, the Western region had lower availability of essential medicines (21, 43, 55). This may be related to the different economic levels of the Eastern, Central, and Western regions. Existing literature showed that, compared with the developed Eastern region, the Western region has insufficient health resources and a lack of high-quality essential medicines (61), which indicated the inequality in the allocation of health resources and the utilization of health services in China (62). Notably, we found that the availability of essential medicines increased moderately in the Western region, but reduced slightly in the Eastern region and the Central region from 2015 to 2019 compared with that from 2009 to 2014. The investigation is needed to shed light on the reasons for the unwanted changes, and tailored measures are warranted to reverse the downward trend of the availability of essential medicines in more developed regions.

Finally, none ATC categories except one reached the fairly high (>50%) availability rate in most recent years, indicating systematic challenges for the NEMS. And the three categories of essential medicines with the lowest availability were similar during two periods, which were nervous system, systemic hormonal preparations, excl. sex hormones and insulins, and antiparasitic products, insecticides and repellents. There may be several reasons for this. First, it may be related to the imperfect pricing mechanism and procurement and distribution system of these medicines (51, 52). Second, pharmacies may unable to sell some medicines due to their small sale volumes, such as antiparasitic products, insecticides and repellents, which may be related to the reduced prevalence of such diseases in the Chinese population (53). Third, some medicines for the nervous system, such as diazepam, are not available in private pharmacies, because it is under special management and patients must purchase them with psychiatrist specialists' prescriptions (17).

Our study has some strengths. First, this is the first systematic review and meta-analysis to comprehensively evaluate the availability of essential medicines in China, which is one of the critical pillars of the healthcare system. Second, methodologically, this systematic review was conducted following consolidated standards including the extensiveness of search strategies, rigor in study selection criteria, extraction of relevant information, and data analysis, which ensured the comprehensiveness and robustness of research findings. Third, the research findings shed lights on the availability of essential medicines in China in aspects of its secular trend, regional distribution, and that by ATC categories in the last decade, which may have important implications for future research and pharmaceutical policy-making toward the goal of universal access to medicines.

Our study has several limitations. Firstly, due to the limited number of included studies, we were unable to perform more detailed analysis to estimate the annual availability of essential medicines. Secondly, the accuracy of pooled estimates of availability of essential medicines may be influenced by varied types of investigated essential medicines involved in primary studies, though we have used pooled estimates based on ATC categories. Thirdly, the WHO/HAI survey methodology required medicines with a specific dosage and form, which may result in underestimates of the availability of some medicines, for other forms and dosages might be available in the pharmaceutical market in China. Finally, though we have systematically searched important databases, websites, and published reviews, there may still be gray literature that has not been included.

## 5. Conclusion

The availability of essential medicines in China is still low compared with the WHO's availability goal, has not changed much in the last decade, is unequal across regions, and lacks data for half of provinces. For research, more studies are warranted, to reveal the reasons and mechanisms of the low availability of essential medicines and facilitate targeted policy-making. A unified investigation method and a standardized list of investigated essential medicines should be formulated according to the healthcare need of Chinese population to promote comparison between studies. For policy-making, the monitoring system of the availability of essential medicines is to be strengthened to enable long-term surveillance, especially in provinces where the data has been missing, as a research and policy priority to enable benchmarking and dedicated efforts for improvement. Meanwhile, joint efforts from all stakeholders including health commission, regulators on drugs, health insurance, pharmaceutical industry, and hospitals are warranted to improve the availability of essential medicines in China toward the universal health coverage target.

## Data availability statement

The original contributions presented in the study are included in the article/Supplementary material, further inquiries can be directed to the corresponding author.

## Author contributions

LZh, KZ, IC, and DL contributed to the conception and designate the study. MZ and KZ participated in drafting and writing the review. MZ, ZL, KZ, DL, YS, ZC, XC, and BL participated in the formulation of retrieval strategies. MZ, ZL, and KZ participated in study selection, data acquisition, and quality assessment. MZ, XW, HL, YJ, YT, SZ, and LZe participated in the data analysis and drawing of tables and figures. All authors contributed to the critical revision of the manuscript and approved the final manuscript.
