# Supplementary material for: Availability of essential medicines, progress and regional distribution in China: a systematic review and meta-analysis
Source: Front Public Health. 2023 Apr 25;11:1149838. doi: 10.3389/fpubh.2023.1149838 (PMC10167309; doi:10.3389/fpubh.2023.1149838)
Supplement: Supplementary file 1 [file Table_1.DOCX]

Supplementary Material

Availability of essential medicines, progress and regional distribution in China: a systematic review and meta-analysis

Miao Zhang^1,2,3,4,5†^, Kun Zou^1,2,3,4†^, Zheng Liu^1,2,3,4,6^, Dan Liu^1,2,3,4,5^, Xiuli Wang^7^, Yuqing Shi^1,2,3,4,5^, Zhe Chen^1,2,3,4,5^, Xiao Cheng^1,2,3,4^, Bingchen Lang^1,2,3,4^, Hailong Li^1,2,3,4^, Linan Zeng^1,2,3,4^, Yong Tang^8^, Shaoyang Zhao^8^, Yongmu, Jiang^8^, Imti Choonara^9^, Lingli Zhang^1,2,3,4,10*^

*** Correspondence:** Lingli Zhang: zhanglingli@scu.edu.cn

# **Supplementary Table 1.** Search strategy in PubMed

| #1 | Essential medicine [Mesh] OR "essential drug*"[Title/Abstract] OR "essential medicine*"[Title/Abstract] OR "essential medication"[Title/Abstract] |
| --- | --- |
| #2 | "China"[Title/Abstract] OR "Chinese"[Title/Abstract] |
| #3 | #1 AND #2 |

# Supplementary Table 2. List of essential medicines investigated in included studies

| ATC | List of essential medicines |
| --- | --- |
| A | Acarbose; Atropine; Calcium Gluconate; Glbibenclamide; Gliclazide; Glimepiride; Glipizide; Insulin; Insulin Glargine; Magnesium Sulfate; Metformin; Omeprazole; Ondansetron; Pioglitazone; Ranitidine; Repaglinide; Vitamin B6 |
| B | Aspirin; Clopidogrel; Folic Acid; Heparin; Hydroxyethyl Starch; Mannitol; Urokinase |
| C | Amiloride; Amlodipine; Atenolol; Atorvastatin; Bisoprolol; Captopril; Digoxin; Diltiazem; Enalapril; Felodipine; Furosemide; Hydrochlorothiazide; Indapamide; Isosorbide Dinitrate; Isosorbide Mononitrate; Labetalol; Levamlodipine; Lisinopril; Losartan; Lovastatin; Metoprolol; Nifedipine; Nimodipine; Nitrendipine; Nitroglycerin; Phentolamine; Prazosin; Propranolol; Simvastatin; Sodium Nitroprusside; Spironolactone; Telmisartan; Triamterene; Urapidil; Valsartan; Valsartan and Amlodipine; Verapamil |
| D | Calamine; Miconazole |
| G | Miconazole; Nystatin |
| H | Dexamethasone; Hydrocortisone; Levothyroxine; Prednisone; Propylthiouracil |
| J | Aciclovir; Amikacin; Amoxicillin; Amoxicillin and Clavulanate Potassium; Ampicillin; Azithromycin; Benzathine Benzylpenicillin; Benzylpenicillin; Cefalexin; Cefazolin; Ceftazidime; Ceftriaxone; Cefuroxime; Chloramphenicol; Ciprofloxacin; Clarithromycin; Clindamycin; Compound Sulfamethoxazole; Doxycycline; Erythromycin; Fluconazole; Gentamycin; Ifosfamide; Isoniazid; Levofloxacin; Linezolid; Metronidazole; Moxifloxacin; Mupirocin; Nitrofurantoin; Norfloxacin; Oseltamivir; Oxacillin; Piperacillin; Piperacillin Sodium and Tazobactam; Promethazine; Rifampicin; Sulfadiazine; Tinidazole; Vancomycin |
| L | Bleomycin; Capecitabine; Carboplatin; Cisplatin; Cyclophosphamide; Cytarabine; Docetaxel; Doxorubicin; Etoposide; Fluorouracil; Gefitinib; Gemcitabine; Ifosfamide; Imatinib; Irinotecan; Methotrexate; Oxaliplatin; Paclitaxel; Pemetrexed; Vincristine; Vinorelbine |
| M | Allopurinol; Ibuprofen; Sodium Diclofenac |
| N | Amitriptyline; Carbamazepine; Citicoline Sodium; Diazepam; Fluoxetine; Morphine; Paracetamol; Phenobarbital; Phenytoin Sodium; Sertraline; Sodium Valproate |
| P | Albendazole; Mebendazole |
| R | Aminophylline; Bromhexine; Cetirizine; Chlorphenamine; Compound Liquorice; Diphenhydramine; Loratadine; Salbutamol |
| S | Mupirocin; Ofloxacin; Sodium Diclofenac |
| V | Calcium Folinate; Protamine |

NR: Not reported. A: Alimentary tract and metabolism; B: Blood and blood forming organs; C: Cardiovascular system; D: Dermatologicals; G: Genito urinary system and sex hormones; H: Systemic hormonal preparations, excl. sex hormones and insulins; J: Antiinfectives for systemic use; L: Antineoplastic and immunomodulating agents; M: Musculo-skeletal system; N: Nervous system; P: Antiparasitic products, insecticides and repellents; R: Respiratory system; S: Sensory organs: V: Various.

# Supplementary Table 3. The risk of bias of included studies

| Included studies | 1. Was the sample frame appropriate to address the target population? | 2. Were study participants sampled in an appropriate way? | 3. Was the sample size adequate? | 4. Were the study subjects and the setting described in detail? | 5. Was the data analysis conducted with sufficient coverage of the identified sample? | 6. Were valid methods used for the identification of the condition? | 7. Was the condition measured in a standard, reliable way for all participants? | 8. Was there appropriate statistical analysis? | 9. Was the response rate adequate, and if not, was the low response rate managed appropriately? | Overall | Quality |
| --- | --- | --- | --- | --- | --- | --- | --- | --- | --- | --- | --- |
| Zhu 2021 | Y | U | Y | Y | N | Y | U | Y | Y | 6 | moderate |
| Wang 2021 | Y | U | Y | Y | Y | Y | Y | Y | Y | 8 | high |
| Zhang 2020 | N | Y | Y | Y | Y | Y | U | Y | U | 6 | moderate |
| Wang 2020 | Y | N | Y | Y | Y | Y | Y | Y | Y | 8 | high |
| Dai 2020 | N | N | Y | Y | Y | Y | N | Y | U | 5 | moderate |
| Zhang 2020 | Y | Y | Y | Y | N | N | N | Y | Y | 6 | moderate |
| Dong 2020 | Y | Y | Y | Y | Y | Y | Y | N | Y | 8 | high |
| Yang 2020 | Y | Y | Y | Y | Y | Y | Y | Y | Y | 9 | high |
| Xu 2020 | Y | Y | Y | Y | Y | Y | Y | Y | Y | 9 | high |
| Jiang 2019 | Y | Y | Y | Y | Y | Y | N | Y | Y | 8 | high |
| Li 2019 | Y | U | Y | Y | N | Y | Y | Y | Y | 7 | high |
| Wei 2019 | N | N | Y | Y | Y | Y | Y | Y | Y | 7 | high |
| Zhu 2019 | Y | Y | Y | Y | Y | Y | Y | Y | Y | 9 | high |
| Yang 2019 | Y | Y | Y | Y | Y | Y | Y | Y | Y | 9 | high |
| Sun 2018 | Y | Y | Y | Y | Y | Y | Y | Y | Y | 9 | high |
| Xi 2018 | N | U | N | Y | N | Y | Y | Y | Y | 5 | moderate |
| Li 2018 | N | Y | Y | Y | N | Y | Y | Y | Y | 7 | high |
| Gong 2018 | Y | Y | Y | Y | Y | Y | U | Y | Y | 8 | high |
| Wu 2018 | N | U | Y | Y | Y | Y | Y | Y | Y | 7 | high |
| Guan 2018 | Y | Y | Y | Y | N | Y | Y | Y | Y | 8 | high |
| Song 2018 | Y | Y | Y | Y | N | Y | Y | Y | Y | 8 | high |
| Su 2017 | Y | U | Y | Y | N | N | Y | Y | Y | 6 | moderate |
| Liu 2017 | Y | Y | Y | Y | Y | Y | U | Y | Y | 8 | high |
| Xie 2016 | Y | U | Y | Y | Y | Y | U | Y | U | 6 | moderate |
| Shang 2016 | Y | N | Y | Y | Y | Y | Y | N | Y | 7 | high |
| Wu 2016 | Y | Y | Y | Y | Y | Y | Y | Y | Y | 9 | high |
| Zhang 2015 | Y | Y | Y | Y | N | Y | U | Y | Y | 7 | high |
| Xi 2015 | Y | Y | Y | Y | Y | Y | Y | Y | Y | 9 | high |
| Jiang 2015 | Y | U | Y | Y | Y | Y | U | N | Y | 6 | moderate |
| Wang 2014 | Y | Y | Y | Y | Y | Y | Y | Y | Y | 9 | high |
| Wang 2014 | Y | N | Y | Y | Y | Y | Y | Y | Y | 8 | high |
| Jiang 2013 | Y | N | Y | Y | Y | Y | Y | Y | U | 7 | high |
| Guan 2013 | Y | N | Y | Y | N | Y | N | Y | Y | 6 | moderate |
| Yan 2013 | Y | Y | Y | N | Y | Y | Y | N | U | 6 | moderate |
| Li 2012 | Y | Y | Y | Y | Y | Y | N | N | Y | 7 | high |
| Li 2011 | Y | Y | Y | Y | N | Y | Y | Y | Y | 8 | high |

Y: Yes; N: No; U: unclear

# Supplementary Table 4. The availability of essential medicines from 2009 to 2019

| ATC | A | B | C | D | G | H | J | L | M | N | P | R | S | V | Overall |
| --- | --- | --- | --- | --- | --- | --- | --- | --- | --- | --- | --- | --- | --- | --- | --- |
| Nationwide | 43.2% | 61.9% | 36.2% | 35.9% | 45.4% | 17.2% | 22.2% | 23.0% | 22.5% | 11.3% | 20.7% | 34.0% | 19.7% | 14.4% | 28.8% |
| 95% CI | 39.4%-47.0% | 51.0%-72.3% | 33.3%-39.2% | 23.4%-49.4% | 38.5%-52.4% | 7.9%-28.7% | 19.5%-25.0% | 16.5%-30.1% | 16.1%-29.6% | 9.0%-13.7% | 12.9%-29.6% | 28.8%-39.5% | 10.3%-30.9% | 1.3%-36.8% | 27.5%-30.1% |
| Eastern region | 49.8% | 59.6% | 51.8% | 21.6% | / | 13.2% | 22.2% | 22.3% | 21.2% | 16.1% | 15.6% | 35.1% | 44.6% | 14.4% | 33.8% |
| 95% CI | 44.3%-55.3% | 46.2%-72.3% | 47.5%-56.0% | 7.7%-39.5% | / | 4.7%-24.4% | 18.2%-26.5% | 15.6%-29.9% | 12.2%-31.9% | 11.3%-21.6% | 1.7%-36.5% | 26.4%-44.4% | 26.6%-63.3% | 1.3%-36.8% | 31.6%-36.1% |
| Beijing | 79.9% | / | 77.2% | / | / | / | / | / | / | / | / | / | / | / | 78.2% |
| 95% CI | 73.6%-85.5% | / | 72.1%-81.9% | / | / | / | / | / | / | / | / | / | / | / | 74.3%-81.9% |
| Liaoning | 0.34.6% | 58.4% | 43.3% | / | / | / | 36.0% | / | / | 63.4% | / | 43% | / | 24.4% | 44.1% |
| 95% CI | 18.5%-52.8% | 38.3%-77.2% | 29.7%-57.4% | / | / | / | 21.4%-52.0% | / | / | 48.3%-77.3% | / | 28%-71.0% | / | 0-71.1% | 36.7%-51.7% |
| Shanghai | 69.4% | 100.0% | 62.5% | / | / | / | 28.8% | / | / | 11.5% | 0 | 74.4% | 54.0% | / | 49.2% |
| 95% CI | 38.1%-94.4% | 93.6%-100% | 43.2%-80.3% | / | / | / | 8.5%-53.4% | / | / | 1.2%-27.2% | 0-6.4% | 35.3%-99.8% | 0-100.0% | / | 37.5%-61.0% |
| Jiangsu | 24.1% | 43.7% | 36.3% | 21.6% | / | 10.8% | 19.3% | 22.3% | 12.8% | 11.2% | / | 27.0% | 44.2% | 8.2% | 22.2% |
| 95% CI | 16.5%-32.6% | 17.3%-71.9% | 22.9%-50.7% | 7.7%-39.5% | / | 3.0%-21.5% | 14.3%-24.7% | 15.6%-29.9% | 4.4%-24.3% | 5.9%-17.6% | / | 14.8%-40.9% | 25.2%-64.1% | 0-28.9% | 19.4%-25.1% |
| Zhejiang | 52.7% | 51.6% | 30.7% | / | / | / | 14.5% | / | 9.1% | 13.4% | 25.4% | 29.2% | 26.8 | / | 24.7% |
| 95% CI | 39.4%-65.7% | 18.9%-83.6% | 21.0%-41.3% | / | / | / | 9.0%-20.9% | / | 0-31.8% | 5.0%-24.6% | 3.8%-56.1% | 14.0%-47.2% | 4.0%-58.8% | / | 20.0%-29.6% |
| Shandong | / | / | / | / | / | / | / | / | / | / | / | / | / | / | 24.5% |
| 95% CI | / | / | / | / | / | / | / | / | / | / | / | / | / | / | 12.5%-38.7% |
| Guangdong | 55.0% | 53.6% | 36.7% | / | / | 48.2% | 34.5% | / | 45.5% | 22.4% | / | 35.5% | / | / | 36.0% |
| 95% CI | 40.6%-69.0% | 40.2%-66.7% | 16.6%-59.4% | / | / | 34.9%-61.5% | 24.2%-45.6% | / | 36.2%-55.0% | 9.6%-38.3% | / | 13.9%-56.5% | / | / | 29.6%-42.8% |
| Tianjin  Hebei  Fujian  Hainan | / | | | | | | | | | | | | | | |
| Central region | 33.8% | 82.1% | 39.9% | / | 37.1% | 36.0% | 34.2% | 11.2% | 52.4% | 14.4% | 27.3% | 38.6% | 66.4% | / | 34.5% |
| 95% CI | 19.9%-49.3% | 77.4%-86.3% | 27.3%-53.2% | / | 29.1%-45.5% | 30.5%-41.7% | 26.4%-42.4% | 6.5%-17.5% | 32.2%-72.2% | 7.2%-23.4% | 21.2%-33.8% | 31.0%-46.5% | 58.3%-73.9% | / | 30.6%-38.5% |
| Anhui | 33.3% | 80.4% | 31.3% | / | 37.1% | 36.0% | 43.9% | 11.2% | 68.9% | 6.2% | 32.9% | 34.4% | / | / | 32.3% |
| 95% CI | 11.2%-60.3% | 73.0%-86.6% | 15.1%-50.2% | / | 29.1%-45.5% | 30.5%-41.7% | 20.5%-68.9% | 6.5%-17.5% | 63.4%-74.1% | 1.2%-14.3% | 25.2%-41.2% | 25.8%-43.4% | / | / | 23.5%-41.9% |
| Jiangxi | / | / | / | / | / | / | / | / | / | / | / | / | / | / | 25.0% |
| 95% CI | / | / | / | / | / | / | / | / | / | / | / | / | / | / | 0-79.3% |
| Henan | / | / | / | / | / | / | / | / | / | / | / | / | / | / | 23.7% |
| 95% CI | / | / | / | / | / | / | / | / | / | / | / | / | / | / | 6.3%-46.4% |
| Hubei | 23.1% | / | / | / | / | / | 28.3% | / | / | / | / | / | / | / | 28.3% |
| 95% CI | 9.4%-40.0% | / | / | / | / | / | 19.3%-38.1% | / | / | / | / | / | / | / | 21.7%-35.4% |
| Heilongjiang  Jilin  Shanxi  Hunan | / | | | | | | | | | | | | | | |
| Western region | 38.9% | 82.3% | 19.8% | 43.8% | / | / | 13.0% | / | 12.8% | 5.9% | 24.1% | 30.4% | 2.5% | / | 19.8% |
| 95% CI | 33.4%-44.4% | 72.1%-90.0% | 15.8%-24.0% | 26.9%-61.4% | / | / | 10.0%-16.3% | / | 5.7%-22.0% | 4.1%-8.0% | 13.2%-37.0% | 22.7%-38.6% | 0.2%-6.5% | / | 18.1%-21.5% |
| Yunnan | / | / | / | / | / | / | / | / | / | / | / | / | / | / | 19.4% |
| 95% CI | / | / | / | / | / | / | / | / | / | / | / | / | / | / | 6.3%-37.2% |
| Shaanxi | 39.2% | 82.3% | 20.0% | 43.8% | / | / | 13.0% | / | 11.6% | 6.0% | 25.0% | 30.0% | 2.5% | / | 19.8% |
| 95% CI | 33.8%-44.8% | 72.1%-90% | 16.0%-24.3% | 26.9%-61.4% | / | / | 10.0%-16.3% | / | 4.8%-20.7% | 4.1%-8.1% | 13.5%-38.5% | 22.2%-38.4% | 0.2%-6.5% | / | 18.0%-21.6% |
| Ningxia | / | / | / | / | / | / | / | / | / | / | / | / | / | / | 50.0% |
| 95% CI | / | / | / | / | / | / | / | / | / | / | / | / | / | / | 3.0%-97.0% |
| Nei Monggol  Guangxi  Chongqing  Sichuan  Guizhou  Gansu  Qinghai  Xinjiang  Xizang | / | | | | | | | | | | | | | | |

ATC: Anatomical Therapeutic Chemical; 95% CI: 95 % confidence interval; /: Provinces, municipalities and autonomous regions either did not conduct the availability of essential medicines or the data were not available; A: Alimentary tract and metabolism; B: Blood and blood forming organs; C: Cardiovascular system; D: Dermatologicals; G: Genito urinary system and sex hormones; H: Systemic hormonal preparations, excl. sex hormones and insulins; J: Antiinfectives for systemic use; L: Antineoplastic and immunomodulating agents; M: Musculo-skeletal system; N: Nervous system; P: Antiparasitic products, insecticides and repellents; R: Respiratory system; S: Sensory organs: V: Various.

# Supplementary Table 5. The availability of essential medicines from 2009 to 2014

| ATC | A | B | C | D | G | H | J | L | M | N | P | R | S | V | Overall |
| --- | --- | --- | --- | --- | --- | --- | --- | --- | --- | --- | --- | --- | --- | --- | --- |
| Nationwide | 49.7% | 77.7% | 37.8% | 43.8% | / | 15.9% | 22.3% | 24.1% | 19.6% | 10.6% | 25.0% | 32.8% | 17.2% | 9.5% | 29.4% |
| 95% CI | 44.2%-55.3% | 69.9%-84.8% | 33.7%-42.0% | 26.9%-61.4% | / | 2.8%-34.6% | 18.4%-26.4% | 14.0%-35.8% | 12.3%-28.0% | 8.1%-13.4% | 13.5%-38.5% | 25.7%-40.2% | 7.0%-30.5% | 4.3%-16.3% | 27.5%-31.3% |
| Eastern region | 66.1% | 69.6% | 67.1% | / | / | 15.9% | 34.1% | 24.1% | 44.1% | 19.3% | / | 40.2% | 65.7% | 9.5% | 47.3% |
| 95% CI | 60.0%-71.9% | 52.6%-84.5% | 62.3%-71.7% | / | / | 2.8%-34.6% | 24.1%-44.8% | 14.0%-35.8% | 37.4%-51.0% | 11.4%-28.5% | / | 23.7%-57.8% | 45.8%-83.4% | 4.3%-16.3% | 43.7%-50.8% |
| Beijing | 79.9% | / | 77.2% | / | / | / | / | / | / | / | / | / | / | / | 78.2% |
| 95% CI | 73.6%-85.5% | / | 72.1%-81.9% | / | / | / | / | / | / | / | / | / | / | / | 74.3%-81.9% |
| Jiangsu | 37.0% | 69.8% | 45.6% |  |  | 11.0% | 22.0% | 24.1% |  | 13.3% |  | 39.7% | 59.2% | 9.5% | 29.5% |
| 95% CI | 19.7%-55.9% | 48.6%-87.7% | 29.5%-62.1% |  |  | 0.4%-28.9% | 10.6%-35.6% | 14.0%-35.8% |  | 3.7%-26.3% |  | 11.4%-71.7% | 42.8%-74.7% | 4.3%-16.3% | 24.0%-35.4% |
| Guangdong | 55.0% | 53.6% | 36.7% | / | / | 48.2% | 34.5% | / | 45.5% | 22.4% | / | 35.5% | / | / | 36.0% |
| 95% CI | 40.6%-69.0% | 40.2%-66.7% | 16.6%-59.4% | / | / | 34.9%-61.5% | 24.2%-45.6% | / | 36.2%-55.0% | 9.6%-38.3% | / | 13.9%-56.5% | / | / | 29.6%-42.8% |
| Tianjin  Hebei  Liaoning  Shanghai  Zhejiang  Fujian  Shandong  Hainan | / | | | | | | | | | | | | | | |
| Central region | 67.9% | 83.6% | 60.5% | / | / | / | 73.3% | / | 32.2% | 26.0% | / | 46.7% | 66.4% | / | 57.9% |
| 95% CI | 43.9%-87.8% | 76.7%-89.1% | 48.3%-72.1% | / | / | / | 62.1%-83.2% | / | 24.9%-40.3% | 12.5%-42.4% | / | 38.6%-55.0% | 58.3%-73.9% | / | 51.8%-63.8% |
| Heilongjiang  Jilin  Shaanxi  Anhui  Jiangxi  Henan  Hubei  Hunan | / | | | | | | | | | | | | | | |
| Western region | 30.8% | 82.3% | 20.0% | 43.8% | / | / | 13.0% | / | 11.6% | 6.0% | 25.0% | 30.0% | 2.5% | / | 17.1% |
| 95% CI | 23.5%-38.5% | 72.1%-90.0% | 16.0%-24.3% | 26.9%-61.4% | / | / | 10.0%-16.3% | / | 4.8%-20.7% | 4.1%-8.1% | 13.5%-38.5% | 22.2%-38.4% | 0.2%-6.5% | / | 15.3%-18.9% |
| Shaanxi | 28.6% | / | 18.7% | 43.8% | / | / | 11.3% | / | 10.1% | 5.2% | 25.0% | 29.2% | 1.3% | / | 15.4% |
| 95% CI | 21.6%-36.2% | / | 14.9%-22.9% | 26.9%-61.4% | / | / | 8.6%-14.4% | / | 3.8%-18.7% | 3.5%-7.1% | 13.5%-38.5% | 21.6%-37.6% | 0.2%-3.1% | / | 13.6%-17.2% |
| Nei Monggol  Guangxi  Chongqing  Sichuan  Guizhou  Yunnan  Xizang  Gansu  Qinghai  Ningxia  Xinjiang | / | | | | | | | | | | | | | | |

ATC: Anatomical Therapeutic Chemical; 95% CI: 95 % confidence interval; /: Provinces, municipalities and autonomous regions either did not conduct the availability of essential medicines or the data were not available; A: Alimentary tract and metabolism; B: Blood and blood forming organs; C: Cardiovascular system; D: Dermatologicals; G: Genito urinary system and sex hormones; H: Systemic hormonal preparations, excl. sex hormones and insulins; J: Antiinfectives for systemic use; L: Antineoplastic and immunomodulating agents; M: Musculo-skeletal system; N: Nervous system; P: Antiparasitic products, insecticides and repellents; R: Respiratory system; S: Sensory organs: V: Various.

# Supplementary Table 6. The availability of essential medicines from 2015 to 2019

| ATC | A | B | C | D | G | H | J | L | M | N | P | R | S | V | Overall |
| --- | --- | --- | --- | --- | --- | --- | --- | --- | --- | --- | --- | --- | --- | --- | --- |
| Nationwide | 37.9% | 54.7% | 33.7% | 22.1% | 45.4% | 18.3% | 22.1% | 22.1% | 26.6% | 12.6% | 16.3% | 35.5% | 25.8% | 16.5% | 28.1% |
| 95% CI | 33.4%-42.5% | 40.0%-69.0% | 29.6%-38.0% | 10.1%-37.0% | 38.5%-52.4% | 6.6%-33.5% | 18.8%-25.5% | 14.1%-31.2% | 16.0%-38.7% | 8.4%-17.5% | 6.7%-28.2% | 27.8%-43.6% | 10.9%-43.8% | 0.4%-46.5% | 26.4%-29.9% |
| Eastern region | 34.9% | 56.2% | 38.5% | 21.6% | / | 10.9% | 18.8% | 20.7% | 13.7% | 14.4% | 15.6% | 34.3% | 32.1% | 16.5% | 26.3% |
| 95% CI | 27.3%-42.9% | 40.0%-71.7% | 31.3%-46.0% | 7.7% -39.5% | / | 1.5%-25.8% | 14.9%-22.9% | 12.2%-30.8% | 5.4%-24.4% | 8.5%-21.3% | 1.7%-36.5% | 24.4%-44.8% | 14.3%-52.6% | 0.4%-46.5% | 24.0%-28.7% |
| Liaoning | 34.6% | 58.4% | 43.3% | / | / | / | 36.0% | / | / | 63.4% | / | 49.3% | / | 24.4% | 44.1% |
| 95% CI | 18.5%-52.8% | 38.3%-77.2% | 29.7%-57.4% | / | / | / | 21.4%-52.0% | / | / | 48.3%-77.3% | / | 27.8%-71.0% | / | 0-71.1% | 36.7%-51.7% |
| Shanghai | 69.4% | 100.0% | 62.5% | / | / | / | 28.8% | / | / | 11.5% | 0 | 74.4% | 0.540% | / | 492% |
| 95% CI | 38.1%-94.4% | 93.6%-100.0% | 43.2%-80.3% | / | / | / | 8.5%-53.4% | / | / | 1.2%-27.2% | 0-6.4% | 35.3%-99.8% | 0-100.0% | / | 37.5%-61.0% |
| Jiangsu | 16.2% | 13.2% | 13.1% | 21.6% | / | 10.9% | 18.5% | 20.7% | 12.8% | 10.2% | / | 23.1% | 26.1% | 7.0% | 17.9% |
| 95% CI | 10.2%-23.3% | 0-44.1% | 1.5%-31.8% | 7.7%-39.5% | / | 1.5%-25.8% | 13.2%-24.4% | 12.2%-30.8% | 4.4%-24.3% | 4.4%-17.6% | / | 11.4%-37.1% | 4.5%-56.1% | 2.5%-13.0% | 15.0%-21.0% |
| Zhejiang | 52.7% | 51.6% | 30.7% | / | / | / | 14.5% | / | 9.1% | 13.4% | 25.4% | 29.2% | 26.8% | / | 24.7% |
| 95% CI | 39.4%-65.7% | 18.9%-83.6% | 21.0%-41.3% | / | / | / | 9.0%-20.9% | / | 0-31.8% | 5.0%-24.6% | 3.8%-56.1% | 14.0%-47.2% | 4.0%-58.8% | / | 20.0%-29.6% |
| Shandong | / | / | / | / | / | / | / | / | / | / | / | / | / | / | 24.5% |
| 95% CI | / | / | / | / | / | / | / | / | / | / | / | / | / | / | 12.5%-38.7% |
| Beijing  Tianjin  Hebei  Fujian  Guangdong  Hainan | / | | | | | | | | | | | | | | |
| Central region | 25.6% | 80.4% | 28.3% | / | 37.1% | 36.0% | 29.5% | 11.2% | 59.8% | 8.9% | 27.3% | 36.4% | / | / | 28.8% |
| 95% CI | 12.6%-41.1% | 73.0%-80.6% | 14.3%-45.0% | / | 29.1%-45.5% | 30.5%-41.7% | 21.5%-38.1% | 6.5%-17.5% | 43.3%-75.3% | 3.4%-16.6% | 21.2%-33.8% | 28.5%-44.7% | / | / | 24.4%-33.5% |
| Anhui | 33.3% | 80.4% | 31.3% | / | 37.1% | 36.0% | 43.9% | 11.2% | 68.9% | 6.2% | 32.9% | 34.4% | / | / | 32.3% |
| 95% CI | 11.2%-60.3% | 73.0%-86.6% | 15.1%-50.2% | / | 29.1%-45.5% | 30.5%-41.7% | 20.5%-68.9% | 6.5%-17.5% | 63.4%-74.1% | 1.2%-14.3% | 25.2%-41.2% | 25.8%-43.4% | / | / | 23.5%-41.9% |
| Jiangxi | / | / | / | / | / | / | / | / | / | / | / | / | / | / | 25.0% |
| 95% CI | / | / | / | / | / | / | / | / | / | / | / | / | / | / | 0-79.3% |
| Henan | / | / | / | / | / | / | / | / | / | / | / | / | / | / | 23.7% |
| 95% CI | / | / | / | / | / | / | / | / | / | / | / | / | / | / | 6.3% -46.4% |
| Hubei | 23.1% | / | / | / | / | / | 28.3% | / | / | / | / | / | / | / | 28.3% |
| 95% CI | 9.4%-40.0% | / | / | / | / | / | 19.3%-38.1% | / | / | / | / | / | / | / | 21.7%-35.4% |
| Heilongjiang  Jilin  Shanxi  Hunan | / | | | | | | | | | | | | | | |
| Western region | 45.1% | / | 1.7% | / | / | / | 13.9% | / | 43.6% | 2.9% | 9.1% | 52.7% | / | / | 38.5% |
| 95% CI | 37.6%-52.8% | / | 0-5.5% | / | / | / | 0-43.2% | / | 30.3%-57.7% | 0-17.8% | 3.0%-20.0% | 38.8%-66.3% | / | / | 34.0%-43.1% |
| Yunnan | / | / | / | / | / | / | / | / | / | / | / | / | / | / | 19.4% |
| 95% CI | / | / | / | / | / | / | / | / | / | / | / | / | / | / | 6.3%-37.2% |
| Shaanxi | 45.9% | / | / | / | / | / | / | / | / | / | / | / | / | / | 44.6% |
| 95% CI | 38.2%-53.6% | / | / | / | / | / | / | / | / | / | / | / | / | / | 37.5%-51.8% |
| Ningxia | / | / | / | / | / | / | / | / | / | / | / | / | / | / | 50.0% |
| 95% CI | / | / | / | / | / | / | / | / | / | / | / | / | / | / | 3.0%-97.0% |
| Nei Monggol  Guangxi  Chongqing  Sichuan  Guizhou  Xizang  Gansu  Qinghai  Xinjiang | / | | | | | | | | | | | | | | |

ATC: Anatomical Therapeutic Chemical; 95% CI: 95 % confidence interval; /: Provinces, municipalities and autonomous regions either did not conduct the availability of essential medicines or the data were not available; A: Alimentary tract and metabolism; B: Blood and blood forming organs; C: Cardiovascular system; D: Dermatologicals; G: Genito urinary system and sex hormones; H: Systemic hormonal preparations, excl. sex hormones and insulins; J: Anti-infectives for systemic use; L: Antineoplastic and immunomodulating agents; M: Musculo-skeletal system; N: Nervous system; P: Antiparasitic products, insecticides and repellents; R: Respiratory system; S: Sensory organs: V: Various.

# Supplementary Table 7. PRISMA 2020 checklist

| **Section and Topic** | **Item #** | **Checklist item** | **Location where item is reported** |  |  |  |
| --- | --- | --- | --- | --- | --- | --- |
| **TITLE** | | |  |  |  |  |
| Title | 1 | Identify the report as a systematic review. | Page 1 |  |  |  |
| **ABSTRACT** | | |  |  |  |  |
| Abstract | 2 | See the PRISMA 2020 for Abstracts checklist. | Page 1-2 |  |  |  |
| **INTRODUCTION** | | |  |  |  |  |
| Rationale | 3 | Describe the rationale for the review in the context of existing knowledge. | Page 2-3 |  |  |  |
| Objectives | 4 | Provide an explicit statement of the objective(s) or question(s) the review addresses. | Page 3 |  |  |  |
| **METHODS** | | |  |  |  | |
| Eligibility criteria | 5 | Specify the inclusion and exclusion criteria for the review and how studies were grouped for the syntheses. | Page 3 |  |  |  |
| Information sources | 6 | Specify all databases, registers, websites, organisations, reference lists and other sources searched or consulted to identify studies. Specify the date when each source was last searched or consulted. | Page 3 |  |  |  |
| Search strategy | 7 | Present the full search strategies for all databases, registers and websites, including any filters and limits used. | Page 3, Table S1 |  |  |  |
| Selection process | 8 | Specify the methods used to decide whether a study met the inclusion criteria of the review, including how many reviewers screened each record and each report retrieved, whether they worked independently, and if applicable, details of automation tools used in the process. | Page 3 |  |  |  |
| Data collection process | 9 | Specify the methods used to collect data from reports, including how many reviewers collected data from each report, whether they worked independently, any processes for obtaining or confirming data from study investigators, and if applicable, details of automation tools used in the process. | Page 3 |  |  |  |
| Data items | 10a | List and define all outcomes for which data were sought. Specify whether all results that were compatible with each outcome domain in each study were sought (e.g. for all measures, time points, analyses), and if not, the methods used to decide which results to collect. | Page 3-4 |  |  |  |
|  | 10b | List and define all other variables for which data were sought (e.g. participant and intervention characteristics, funding sources). Describe any assumptions made about any missing or unclear information. | Page 3 |  |  |  |
| Study risk of bias assessment | 11 | Specify the methods used to assess risk of bias in the included studies, including details of the tool(s) used, how many reviewers assessed each study and whether they worked independently, and if applicable, details of automation tools used in the process. | Page 3-4 |  |  |  |
| Effect measures | 12 | Specify for each outcome the effect measure(s) (e.g. risk ratio, mean difference) used in the synthesis or presentation of results. | Page 4 |  |  |  |
| Synthesis methods | 13a | Describe the processes used to decide which studies were eligible for each synthesis (e.g. tabulating the study intervention characteristics and comparing against the planned groups for each synthesis (item #5)). | Page 4 |  |  |  |
|  | 13b | Describe any methods required to prepare the data for presentation or synthesis, such as handling of missing summary statistics, or data conversions. | Page 4 |  |  |  |
|  | 13c | Describe any methods used to tabulate or visually display results of individual studies and syntheses. | Page 4 |  |  |  |
|  | 13d | Describe any methods used to synthesize results and provide a rationale for the choice(s). If meta-analysis was performed, describe the model(s), method(s) to identify the presence and extent of statistical heterogeneity, and software package(s) used. | Page 4 |  |  |  |
|  | 13e | Describe any methods used to explore possible causes of heterogeneity among study results (e.g. subgroup analysis, meta-regression). | Page 4 |  |  |  |
|  | 13f | Describe any sensitivity analyses conducted to assess robustness of the synthesized results. | Not applicable |  |  |  |
| Reporting bias assessment | 14 | Describe any methods used to assess risk of bias due to missing results in a synthesis (arising from reporting biases). | Not applicable |  |  |  |
| Certainty assessment | 15 | Describe any methods used to assess certainty (or confidence) in the body of evidence for an outcome. | Page 4 |  |  |  |
| **RESULTS** | | |  |  |  |  |
| Study selection | 16a | Describe the results of the search and selection process, from the number of records identified in the search to the number of studies included in the review, ideally using a flow diagram. | Page 4, Figure 1 |  |  |  |
|  | 16b | Cite studies that might appear to meet the inclusion criteria, but which were excluded, and explain why they were excluded. | Page 4, Figure 1 |  |  |  |
| Study characteristics | 17 | Cite each included study and present its characteristics. | Page 4, Table 1, Table S2 |  |  |  |
| Risk of bias in studies | 18 | Present assessments of risk of bias for each included study. | Page 5, Table S3 |  |  |  |
| Results of individual studies | 19 | For all outcomes, present, for each study: (a) summary statistics for each group (where appropriate) and (b) an effect estimate and its precision (e.g. confidence/credible interval), ideally using structured tables or plots. | Page 5 |  |  |  |
| Results of syntheses | 20a | For each synthesis, briefly summarise the characteristics and risk of bias among contributing studies. | Page 5-6 |  |  |  |
|  | 20b | Present results of all statistical syntheses conducted. If meta-analysis was done, present for each the summary estimate and its precision (e.g. confidence/credible interval) and measures of statistical heterogeneity. If comparing groups, describe the direction of the effect. | Page 5-6 |  |  |  |
|  | 20c | Present results of all investigations of possible causes of heterogeneity among study results. | Page 5-6 |  |  |  |
|  | 20d | Present results of all sensitivity analyses conducted to assess the robustness of the synthesized results. | Not applicable |  |  |  |
| Reporting biases | 21 | Present assessments of risk of bias due to missing results (arising from reporting biases) for each synthesis assessed. | Not applicable |  |  |  |
| Certainty of evidence | 22 | Present assessments of certainty (or confidence) in the body of evidence for each outcome assessed. | Page 5-6 |  |  |  |
| **DISCUSSION** | | |  |  |  |  |
| Discussion | 23a | Provide a general interpretation of the results in the context of other evidence. | Page 6-7 |  |  |  |
|  | 23b | Discuss any limitations of the evidence included in the review. | Page 7 |  |  |  |
|  | 23c | Discuss any limitations of the review processes used. | Page 7-8 |  |  |  |
|  | 23d | Discuss implications of the results for practice, policy, and future research. | Page 8 |  |  |  |
| **OTHER INFORMATION** | | |  |  |  |  |
| Registration and protocol | 24a | Provide registration information for the review, including register name and registration number, or state that the review was not registered. | Page 3 |  |  |  |
|  | 24b | Indicate where the review protocol can be accessed, or state that a protocol was not prepared. | Page 3 |  |  |  |
|  | 24c | Describe and explain any amendments to information provided at registration or in the protocol. | Not applicable |  |  |  |
| Support | 25 | Describe sources of financial or non-financial support for the review, and the role of the funders or sponsors in the review. | Page 8 |  |  |  |
| Competing interests | 26 | Declare any competing interests of review authors. | Page 8 |  |  |  |
| Availability of data, code and other materials | 27 | Report which of the following are publicly available and where they can be found: template data collection forms; data extracted from included studies; data used for all analyses; analytic code; any other materials used in the review. | Not applicable |  |  |  |
